# Progress in understanding the role of cGAS-STING pathway associated with programmed cell death in intervertebral disc degeneration

**DOI:** 10.1038/s41420-023-01607-7

**Published:** 2023-10-16

**Authors:** Zheng Wang, Xinli Hu, Peng Cui, Chao Kong, Xiaolong Chen, Wei Wang, Shibao Lu

**Affiliations:** 1 Department of Orthopedics, Xuanwu Hospital, Capital Medical University, No. 45 Changchun Street, Xicheng District, Beijing, 100053 China; 2 National Clinical Research Center for Geriatric Diseases, Xuanwu Hospital, Capital Medical University, Beijing, 100053 China

**Keywords:** Cell death, Cell signalling

## Abstract

Nucleus pulposus (NP) inflammatory response can induce intervertebral disc degeneration (IVDD) by causing anabolic and catabolic disequilibrium of the extracellular matrix (ECM). This process is accompanied by the production of endogenous DNAs, then detectable by the DNA sensor cyclic GMP-AMP synthase (cGAS). cGAS recognizes these DNAs and activates the downstream adaptor protein, a stimulator of interferon genes (STING), initiating a cascade of inflammation responses through various cytokines. This evidence implies a crucial role of the cGAS-STING signaling pathway in IVDD. Additionally, it is suggested that this pathway could modulate IVDD progression by regulating apoptosis, autophagy, and pyroptosis. However, a detailed understanding of the role of cGAS-STING pathway in IVDD is still lacking. This review provides a comprehensive summary of recent advances in our understanding of the role of the cGAS-STING pathway in modulating inflammatory response in IVDD. We delve into the connection between the cGAS-STING axis and apoptosis, autophagy, and pyroptosis in IVDD. Furthermore, we discuss the therapeutic potential of targeting the cGAS-STING signaling pathway in IVDD treatment. Overall, this review aims to provide a foundation for future directions in IVDD treatment strategies.

## Facts

1. Cytosolic DNA stimulates the cGAS–STING signaling pathway that initiates type I interferon production and different modes of cell death.
2. Autophagy exerts a dual role in cGAS–STING signaling by targeting STING for degradation.
3. Different modes of cell death are closely related to the cGAS-STING signaling pathway.
4. cGAS–STING signaling pathway connecting inflammation and cell death plays key roles in pathogenesis of intervertebral disc degeneration.

## Open questions

1. What are the mechanisms by which the cGAS–STING induces different modes of cell death?
2. Whether there are intersections that lead to cGAS-STING inducing different modes of cell death?
3. What is the relationship between other modes of cell death (ferroptosis and necroptosis) and cGAS-STING?
4. What are the adverse effects of targeting cGAS–STING signaling in IVDD?

## Introduction

Low back pain (LBP), a leading cause of disability globally with a prevalence rate exceeding 80%, imposes significant economic strain on societies, exceeding $100 billion per year [1, 2]. Intervertebral disc degeneration (IVDD) is the primary culprit behind most instances of LBP, with aging, genetics, biomechanics, and environmental factors contributing to its occurrence. IVDD patients often display impaired nucleus pulposus (NP) cell function and an imbalance in the anabolic and metabolic processes of the extracellular matrix (ECM) [3–5]. Research also suggests that inflammatory responses may play a role in IVDD development [6, 7]. In IVDD, NP cells undergo phenotypic changes, triggering the upregulation of IL-6 and IL-8. This alters the catabolic and anabolic dynamics in the ECM, thus contributing to the progression of IVDD [8]. However, the precise molecular mechanisms and pathways underpinning NP cells inflammation and ECM degradation remain elusive and warrant further investigation [9]. Potential strategies for targeted intervention in these abnormal signals are also needed. The Stimulator of Interferon Genes (STING) was initially identified as a protein interacting with major histocompatibility complex class II molecules, though the implications of this interaction are yet to be fully elucidated [10]. In an endeavor to pinpoint the progenitors of excessive interferon-β (IFN-β) production, Ishikawa and colleagues utilized an expression screening system in 2008. This approach facilitated the detection of proteins that precipitate IFN-β secretion. In the scope of their study, around 5500 human and 9000 murine full-length cDNAs were individually introduced into cells, which housed a luciferase gene under the jurisdiction of the IFNβ promoter. Their systematic analysis culminated in the identification of five genes whose overexpression prompted considerable IFNβ induction. Among these, the authors classified one of the hitherto undefined molecules as STING [11]. Complementary studies utilizing STING-deficient mice subsequently ratified the pivotal function of STING in fostering innate responses to IFNβ stimulation [12]. Later, in 2011, it was found that TANK binding kinase 1 (TBK1), a downstream molecule of STING, phosphorylates IRF3 and the NF-κB pathway [13]. Following this, it was observed that certain DNA sensors and upstream regulators, such as cGAMP and interferon gamma inducible protein 16 (IFI16), could facilitate STING activation [14]. A breakthrough came in 2013 when Wu and Sun discovered that cyclic guanosine monophosphate-adenosine monophosphate (cGAMP) could function as a ligand for STING [15]. They identified a novel protein, “cyclic-GMP-AMP synthase” (cGAS), which could sense cytoplasmic DNA and allows GMP and AMP to synthesize cGAMP [15, 16]. This insight filled a critical knowledge gap in the cGAS-STING signaling pathway. Over the last decade, an increasing number of researchers have been focusing on the cGAS-STING signaling pathway, linking it to various conditions, including inflammation, infection, autoimmune diseases, metabolic disorders, tumors, and IVDD [17–20]. This review will discuss the role of the cGAS-STING signaling pathway in IVDD, emphasizing its connection with apoptosis, autophagy, pyroptosis, and necrosis in IVDD. It will also explore the future prospects of researching the cGAS-STING signaling pathway and its significance in treating IVDD.

## Overview of cGAS-sting signaling pathway

**Fig. 1:**
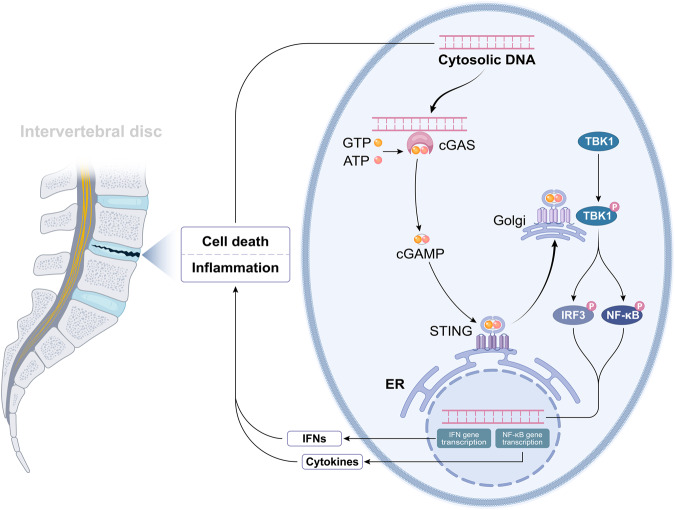
Activation of cGAS-STING signaling pathway mediates the IVDD. In degenerative discs, inflammation and cell death could liberate DNAs aberrantly. cGAS can identify the cytosolic DNA, transform ATP and GTP into the second messenger 2′,3′-cGAMP, and then bind to and enhance the activation of STING in the ER. After STING was transported to the Golgi apparatus, it could activate the downstream signaling pathway of TBK1-IRF3 and -NF-κB, and enhance the activity of IRF3 and NF-κB. With increased activity, IRF3 and NF-κB may enter the nucleus and play a role in elevating the expression of type-I IFN and pro-inflammatory cytokines, respectively.

The presence of pathogen-related molecular patterns (PAMPs) and danger-related molecular patterns (DAMPs), derived from microbial pathogens and abnormal autologous cells, prompts cellular responses [21, 22]. Pattern recognition receptors (PPRs), dispersed widely in mammalian cell membranes, are designed to detect intracellular and extracellular PAMPs and DAMPs, thus initiating the immune response [23, 24]. Over the past two decades, various PPRs have been identified, which include membrane-bound Toll-like receptors (TLRs), C-type lectin receptors (CLRs), cytoplasmic NOD-like receptors (NLRs), and gene I-like receptors (RLRs) [21]. These PRRs are known to mediate the activity of DNA sensors such as TLR9, absent in melanoma 2 (AIM2), and cyclic GMP-AMP (cGAMP) synthase (cGAS) [15, 25, 26]. Among these DNA sensors, TLR-9 has been extensively studied. Located on the endosomal membrane, TLR-9 swiftly triggers an innate immune response upon detection of a potential threat to the host [27]. AIM2, recognized as a cytoplasmic DNA receptor in 2009, can form an inflammasome by binding to the ASC (apoptosis-associated speck-like protein containing a caspase recruitment domain) when it detects the presence of dsDNA, thereby inducing pyroptotic cell death [26, 28]. In 2013, Chen et al. discovered cGAS, which stimulates ATP and GTP to produce a second messenger, cGAMP, instead of directly creating signaling molecules [15]. cGAMP activates the IFN-I signaling pathway upon binding to STING [29]. This review emphasizes the role of cGAS in detecting DAMPs and PAMPs. This ability of cGAS to sense mislocalized DNAs is a critical event in cellular responses to PAMPs and DAMPs. cGAS can recognize DNAs of varying sizes in a sequence-independent manner, with sequences as short as 45 bp being detectable [30]. Larger DNA fragments tend to yield a more robust immune response [31]. Upon DNA binding, cGAS undergoes conformational changes, catalyzing cGAMP synthesis from ATP and GTP [32]. The cGAMP then binds to the adapter protein STING, anchored to the endoplasmic reticulum (ER), thereby leading to a conformational shift and STING activation [31]. The cGAMP-bound STING then migrates from the ER to the Golgi apparatus [33, 34]. STING interacts with TANK-binding kinase 1 (TBK1), culminating in TBK1-mediated STING phosphorylation. The phosphorylated STING subsequently associates with the electro-positive domains of Interferon Regulatory Factor 3 (IRF3), which leads to IRF3 phosphorylation and activation [35]. The phosphorylation of IRF3 induces the translocation of IRF3 from the cytoplasm to the nucleus. Within the nuclear environment, IRF3 targets the Interferon-stimulated response element,specifically within the promoter of the Interferon-stimulated gene 15 (ISG15), thereby enhancing its transcriptional activity and resulting in a robust expression of type I interferon and a series of inflammatory factors [36]. (Fig. 1).

## cGAS-sting signaling pathway in IVDD

The intervertebral disc is composed of a gel-like nucleus pulposus (NP) tissue in the center. NP cells are critical for secreting ECM proteins, which maintain disc height and alleviate axial mechanical pressure on the valve body [37]. In terms of molecular mechanisms, intervertebral disc degeneration (IVDD) primarily manifests through the aging and inflammation of NP cells and the degradation of ECM [38, 39]. Inflammation poses a twofold problem: firstly, it irreversibly damages the vertebral body and endplate structure, and secondly, it impairs blood circulation to the intervertebral disc. This restriction of oxygen and nutrient supply ultimately leads to cell death, a process accompanied by the generation of cytoplasmic DNA [40]. The cGAS/STING signaling pathway plays a key role in the activation of the inflammatory response. Cytoplasmic DNA, recognized by cGAS as a danger signal, leads to downstream STING playing the part of adapter molecules. This triggers TBK1 and IRF3, generating cytokines that stimulate the immune response [41]. Lipopolysaccharide (LPS) has been found to activate the cGAS/STING signaling pathway, leading to the production of various inflammatory factors. This induced inflammatory response can promote the development of IVDD [42, 43]. In a significant study, Su et al. established a close link between the LPS-activated cGAS/STING signaling pathway and IVDD [19]. They suggested that LPS is instrumental in causing massive M1 macrophage infiltration and inflammation, resulting in cell death and the release of free DNAs. This, in turn, activates downstream signals by binding to cGAS, elevating immune responses, and increasing cytokine levels. The culmination of these events-cell death, ECM remodeling-ultimately results in IVDD [19]. Although existing studies indicate that the cGAS-STING signaling pathway can induce intervertebral disc aging, it remains to be seen whether this pathway is independently involved in IVDD caused by chemical or mechanical damage [20]. Interestingly, Ottone et al. reported an opposing effect of the activation of STING on IVDD [44]. They suggested that the cGAS-STING signaling pathway does not significantly contribute to disc aging and degeneration when the structural integrity of the disc remains unharmed. One possible explanation for this contradiction could be that the STING signaling pathway remains dormant in the absence of chemical or traumatic mechanical insult. However, when dsDNA is detected in the presence of injury or diseased tissue, the cGAS-STING signaling pathway could be activated, acting as a major regulator of inflammation [45]. Chen et al. developed an innovative, injectable, and self-healing siRNA delivery hydrogel. They found that minimally invasive hydrogel injection slowed the inflammation and progression of IVDD in rats. The underlying mechanism involves siRNA significantly reducing IVD inflammation and slowing down IVDD by silencing STING expression in NP cells [46]. These studies collectively suggest that the cGAS-STING signaling pathway plays a crucial role in IVDD in the event of intervertebral disc injury, and interventions targeting this pathway could help mitigate IVDD.

## Crosstalk between the cGAS-sting signaling pathway and various types of cell death in IVDD

**Fig. 2:**
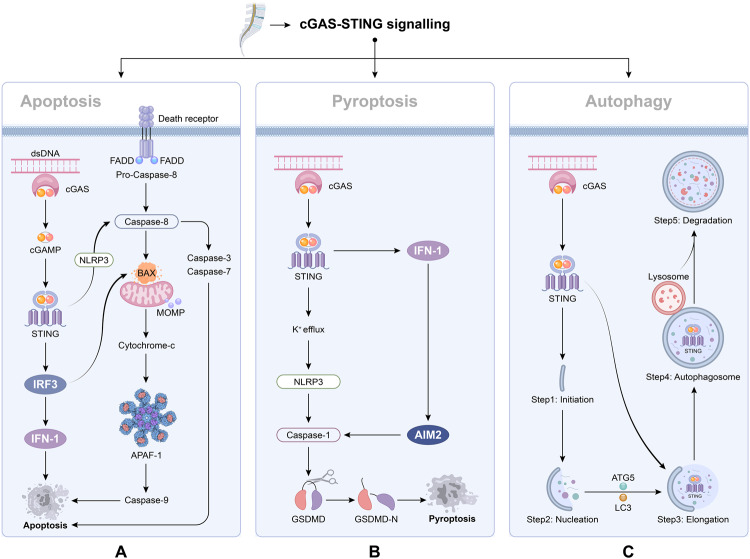
Schematic of association between cGAS-STING signaling pathway and cell death. Herein, impacts of hyperactivated cGAS–STING signaling pathway were summarized, especial its relation with various cell death. **A** Apoptosis is a non-inflammatory process mediated by caspase enzymes, which can be extrinsic or intrinsic. In extrinsic apoptosis, death receptors such as TNFR1 activate the caspase-8, which can cleave the effector caspases-3/7 and accelerate the apoptosis. Intrinsic apoptosis occurs if the mitochondrial outer membrane potential (MOMP) is lost or the BAX-BAK-mediated cytochrome c is released from the mitochondria into the cytosol. The cGAS-STING signaling pathway can induce apoptosis through two pathways. First, cGAS-STING signaling pathway induces apoptosis through NLRP3. STING binding with NLRP3 could enhance the activation of the NLRP 3 inflammasome, which activate caspase-8 leading to extrinsic pathway apoptosis. Second, the cGAS-STING signalling pathway induces apoptosis through IRF3-Bax interaction. Activated IRF3 could bind cytosolic Bax, which results in MOMP and cytochrome c are released to destroy organelles and cause cell death. To sum up, cGAS-STING signaling pathway can induce apoptosis through the intrinsic pathway and extrinsic pathway. **B** In a typical model of pyroptosis, the inflammasome sensor proteins located in cells effectively recognize DAMPs, which leads to NLRP3 oligomerization. NLRP3 then binds to the protein ASC to activate caspase-1, enhancing the activities of IL-1β and IL-18. GSDMD is then cleaved and releases the GSDMD-N domain that forms the membrane pore, which in turn promotes the release of activated IL-1β and IL-18. The relationship between cGAS-STING and pyroptosis is divided into two aspects. On the one hand, activation of STING promoted K^+^ efflux, which decline in cytosolic K^+^, thus activating the classical mode of NLRP3 inflammasome, and then mediates the release of IL-1β and IL-18 to promote pyroptosis. On the other hand, activation of cGAS–STING–IFN1 signaling pathway upregulated the AIM2, thus promoting pyroptosis. **C** Autophagy can be divided into (1) initiation, (2) nucleation, (3) elongation, (4) autophagosome formation, and (5) degradation. cGAS-STING can activate autophagy. After being activated by cGAMP, STING migrates from ER to Golgi apparatus via ERGIC. In ERGIC, STING was involved in autophagy induction. ERGIC containing STING can promote LC3 lipidation and the formation of autophagosomes. Finally, the resulting autophagy then fuses with the lysosome for degradation. It was found that under the mediation of STING, autophagy can effectively remove cytoplasmic DNA after DNA damage and also inhibit STING signaling pathway and its excessive responses, implying that autophagy has a negative feedback effect on the regulation of cGAS-STING signaling pathway.

Cell death, which occurs when cells are damaged and die, is broadly categorized into apoptosis and necrosis. As research deepens, various other cell death modalities, such as autophagy and pyroptosis have been reported [47, 48]. Given the crucial roles the cGAS-STING signaling pathway plays in regulating cell death, numerous researchers have investigated its relationship with cell death [49, 50]. Here, we summarize the connection between the cGAS-STING signaling pathway and cell death in IVDD (Fig. 2).

### Apoptosis

Apoptosis is vital in eliminating infected cells, optimizing the immune system’s activity, supporting organism growth, and maintaining normal cell turnover to uphold homeostasis [51]. Apoptosis can be triggered by either extrinsic or intrinsic pathways. The former involves death ligands (such as TNF, FASL, and TRAIL) binding to TNF superfamily receptors (like TNFR1, FAS, and death receptor 4/5), which then recruit cytoplasmic adapter proteins that activate caspase-8 [52]. This caspase-8 serves as a central protease in death receptor-induced apoptosis, facilitating the activation of caspase-3. On the other hand, the intrinsic pathway can be stimulated by DNA damage, ischemia, and oxidative stress, and is influenced by the mediated action of several members in the Bcl family [53]. The cGAS-STING signaling pathway can sense viral infections, stimulating the production of type 1 IFN, which in turn leads to apoptosis [54, 55]. For instance, IFN-β can activate apoptosis through the intrinsic pathway by down-regulating PI3K/AKT signaling, facilitating the release of cytochrome c, and catalyzing the activation of procaspase 9 [54]. Contrarily, another study proposed that IFN-β induced apoptosis depends on caspase-8 via the extrinsic pathway, and that caspase-8-targeting inhibitors, as opposed to caspase-9-targeting inhibitors, can successfully block IFN-β mediated apoptosis [56]. Furthermore, downregulating IRF3 can weaken the proapoptotic role of the cGAS-STING signaling pathway [57]. Research showed that IRF3 could promote the initiation of an antiviral response and increase the expression of pro-apoptotic genes, expediting apoptosis [58]. Guo et al. demonstrated that the cGAS-STING signaling pathway influences disc degeneration through apoptosis. Overexpressed STING was found to promote the degradation of ECM and apoptosis of NP cells, with a mechanistic study indicating that STING mediated NP cell apoptosis by enhancing the activation of IRF3 [59]. This process was attenuated by silencing the STING, alleviating puncture induced IVDD [59]. Furthermore, the cGAS-STING signaling pathway can activate apoptosis through another route. A study discovered significantly upregulated cGAS, Sting, and NLRP3 mRNA in IVD samples, and showed that EGCG could effectively prevent apoptosis by reducing cGAS/Sting/NLRP3 levels [43]. It was suggested that activating the NLRP3 inflammasome can expedite pyroptosis and apoptosis [60]. In a scenario where the inflammasome is activated but pyroptosis is inhibited, caspase-8 can promote cell death by initiating apoptosis [61, 62]. Thus, the cGAS-STING signaling pathway can induce apoptosis by activating the NLRP3 pathway.

### Pyroptosis

Unlike apoptosis, pyroptosis is a form of programmed cell death typically triggered by an inflammatory response [63]. In general, oligomeric NLRP3 and recruited ASCs initiate the autocatalysis of caspase-1, transforming inactive IL-1β and IL-18 into their active forms. Additionally, the action of active caspase-1 facilitates the conversion of GSDMD into GSDMD-NT, which forms plasma membrane pores to induce pyroptosis [64, 65]. A study by Zhang et al. found that oxidative stress could activate the cGAS-STING axis, triggering NLRP3 inflammasome-mediated pyroptosis in human NP cells in a STING-dependent manner [66]. Interestingly, the mitochondrial permeability transition pore (mPTP) of human NP cells, under oxidative stress stimulation, influences the cytoplasmic escape of mitochondrial DNA, exhibiting some distinctive morphological and functional traits. The use of a specific mPTP inhibitor greatly reduced NLRP3 inflammasome-mediated pyroptosis, along with microenvironmental inflammation and degenerative progression in a rat disc. This underscored the pivotal role of the cGAS-STING-NLRP3 axis and pyroptosis in IVDD [66]. Moreover, Tian et al. indicated that EGCG could thwart apoptosis and inflammation by inhibiting the cGAS/Sting/NLRP3 signaling pathway [43]. The STING-mediated activation of pyroptosis may occur through the stimulation of the NLRP3 inflammasome via K^+^ efflux and membrane perturbation. It is suggested that active STING promotes K^+^ efflux, decreasing cytoplasmic K^+^ concentration and consequently triggering the classical NLRP3 inflammasome activation pattern. This in turn mediates the release of IL-1β and IL-18 in human monocytes, encouraging sterile inflammation and pyroptosis [67]. However, it remains unconfirmed whether the cGAS-STING signaling pathway regulates pyroptosis in IVDD through this mechanism. Numerous studies have focused on the role of the AIM2 inflammasome in the defense mechanism [68, 69]. Elevated AIM2 levels were observed in degenerated discs, correlating with IVDD severity [70]. Inhibition of AIM2 can alleviate H2O2-induced DNA damage and apoptosis in NP cells in vitro and decelerate IDD in vivo. An important question is whether cGAS and AIM2 initiate immune responses simultaneously. Research suggests that activation of the cGAS-STING-IFN1 signaling pathway can amplify AIM2 protein levels, inducing robust immune responses [71]. This indicates that cGAS signaling might precede AIM2 activation. Nevertheless, whether the activation of cGAS-STING-NLRP3 substitutes AIM2 function in response to cytoplasmic DNA in human NP cells is not clear. This leads to another question: does cGAS exhibit higher sensitivity to cytoplasmic DNA compared to AIM2 There is evidence that cGAS–STING shows a superior detection ability than AIM2 in sensing low-level DNAs, highlighting a valuable direction for future research.

### Autophagy

Autophagy is a process of self-digestion within eukaryotic cells. This highly conserved mechanism allows cells to remove damaged organelles, protein aggregates, and invading pathogens [72]. It generally encompasses macroautophagy, microautophagy, and chaperone-mediated autophagy [73]. Macroautophagy (commonly referred to as autophagy) is the most extensively studied form, playing a crucial role in cellular survival, death, and homeostasis. The process entails vesicle nucleation, membrane elongation, autophagosome formation, fusion of autophagosomes with lysosomes to form autolysosomes, and autophagic degradation [74, 75]. The cGAS-STING pathway has been confirmed to influence autophagy regulation. Watson et al. found that extracellular DNA from Mycobacterium tuberculosis could trigger autophagy by enhancing STING activation, suggesting a link between STING and autophagy [76]. In 2017, Bhatelia et al. reported that activation of IRF3 regulated autophagy flux [77]. Moretti et al. provided evidence that STING, present on autophagosomal membranes, senses live Gram-positive bacteria to mediate ER-phagy and the type I IFN response [78]. STING can bind with cGAMP to alter its conformation. The oligomeric STING then migrates from the ER to the Golgi apparatus via the ER-Golgi intermediate compartment (ERGIC). In this process, STING initiates autophagy induction, and ERGIC containing STING promotes LC3 lipidation and the formation of autophagosomes. The resulting autophagy then merges with the lysosome for degradation [79]. Understanding connection of STING to autophagy is vital for studying their crosstalk. Furthermore, Watson et al. proposed that STING-induced selective autophagy is dependent on p62 [80]. Degradation of STING can be observed with the combined action of autophagy, TBK1, and p62, preventing STING-induced immune damage [81]. Conversely, Liu et al. argued that STING-induced autophagy was dependent on ATG5 rather than p62 [82]. Another study found that in the presence of WIPI2 and ATG5, activated STING could lead to LC3 lipidation [79]. These findings confirm that STING is an autophagy substrate and initiator of autophagy induction. However, the protein upon which STING depends to induce autophagy remains debatable. Additionally, autophagy can clear damaged cytoplasmic DNA under mediation of STING, highlighting unique role of autophagy in the STING and IFN-I induced processes. Moreover, upstream and downstream molecules and proteins (cGAMP, cGAS, and TBK1) of the STING signaling can play a role in autophagy activation directly or indirectly. Interestingly, their role in inducing autophagy can inhibit over-response of the STING signal [83, 84]. This finding implies that the STING signal possesses a negative feedback control mechanism. However, the mechanism of this cross-regulatory effect in persistent pathological conditions, especially in IVDD, requires further investigation. To date, only one study has reported the interaction between autophagy and STING in IVDD. Ren et al. found that metformin may suppress the activation of the cGAS-STING signaling pathway by enhancing autophagy, thereby inhibiting IVDD [85]. This implies that further research into the interaction mechanism between STING and autophagy in IVDD is warranted.

## cGAS-sting signaling pathway: a potential therapeutic target in IVDD

Overall, the existing literature emphasizes the critical role of the cGAS-STING pathway in IVDD, particularly its connection with inflammation. Hence, further research on the regulatory mechanisms related to the cGAS-STING signaling pathway is necessary. It may provide valuable insights for the development of promising targeted therapies. In recent years, numerous studies have centered around the development of inhibitors or agonists targeting the cGAS-STING-TBK1 pathway. Although most drugs are designed for treating tumors and central nervous system disorders, they can offer a reference for IVDD treatment (Table 1).

**Table 1:** Overview of cGAS-STING signaling pathway inhibitors.

Target	Inhibitor(s)	Molecular mechanism	Models	Reference
cGAS	Antimalarial drugs (primaquine, chloroquine, hydroxychloroquine, etc.)	Disrupting cGAS/DNA binding complex interface via interacting with DNA	THP1 cells	[86]
cGAS	RU.521	Exerting an inhibitory effect on ATP and GTP catalytic active sites on cGAS, affecting the production of cGAMP	RAW macrophage cells	[87, 88]
cGAS	Suramin, oligodeoxynucleotides A151, and X6	Competitive inhibition of DNA binding sites of cGAS.	Human monocytes and THP1 cells	[89, 90]
cGAS	Aspirin	Acetylating cGAS at three lysine residues and blocking the activity of cGAS	THP-1, human PBMCs, and mouse bone marrow cells	[91]
cGAS	EGCG	EGCG disrupted existing G3BP1-cGAS complexes and inhibited the activation of cGAS induced by DNAs, inhibiting the generation of IFN due to DNA.	U937 cells and Trex1 − /− bone marrow cells	[92]
STING	Astin C	Binding to the C-terminal activation pocket of STING and competitively displacing CDNs to block the recruitment of IRF3 onto the STING signalosome.	HEK293 cells	[93]
STING	Nitrofuran derivatives (C-176, C-178, C-170, C-171, and H-151)	Suppressing the STING clustering caused by palmitoylation by modifying the Cys91 residue in a covalent way.	HEK293T cells	[95]
STING	Tetradroisoquinolone acetic acid	Displacing cGAMP from its binding site on STING	Not mentioned	[96]
STING	Amlexanox	Blocking the full activation of STING by inhibiting the STING at Ser366 induced by TBK1	Mouse lungs	[98–100]
Cytosolic DNA	Metformin and Rapamycin	Autophagy can decrease the load of cytoplasmic DNA	Nucleus pulposus cells and NIH3T3 cells and MRC-5 cells	[85, 104]

### Inhibitors of cGAS

cGAS plays a significant role in producing the second messenger cGAMP, essential for STING signaling. Thus, it is a common target when treating cGAS and STING-dependent inflammatory diseases. A research team identified antimalarial drugs (including quinacrine and hydroxychloroquine) that inhibit cGAS activity from cGAS-dsDNA crystal structures in mice. These drugs bind to cGAS/dsDNA dimers specifically, leading to a change in the stability of the cGAS/dsDNA complex and inhibition of cGAS activation [86]. Subsequent research showed that antimalarial drugs inhibited IFNβ expression, cGAMP production, and cytokines (IL-6 and TNFα). RU.521, another inhibitor, does not directly affect dsDNA binding but interferes with cGAS activity. It operates primarily by occupying the catalytic site of cGAS, weakening its affinity for ATP and GTP. However, it exhibits potent mouse activity but weak inhibitory activity in recombinant human cGAS [87]. This discrepancy might be due to the low similarity of amino acids in human and mouse cGAS (about 60%). Another study found that small molecule forms of G108 and G150 could bind with human cGAS to produce co-crystallization, occupying the ATP- and GTP-binding active site to suppress cGAS activity, similarly to RU.521, in primary human macrophages [88]. Furthermore, Suramin, oligodeoxynucleotides A151, and X6 inhibited cGAS activation competitively through dsDNA displacement from cGAS [89, 90]. Moreover, post-translational modifications such as acetylation, ubiquitination, and sumoylation of cGAS could similarly offer critical potential regulatory targets to limit cGAS activation. Therapeutics that target these cGAS post-translational modifications can modulate cGAS activity. For instance, aspirin was found to inhibit cGAS activity by acetylating its three lysine residues [91]. In an Aicardi-Goutières syndrome (AGS) mouse model, Liu et al. demonstrated the critical role of G3BP1, a protein known to regulate RNA stress response, in the regulation of the cGAS-mediated DNA-sensing pathway. They found that G3BP1 forms a large complex with cGAS and promotes cGAS oligomerization [92]. Notably, epigallocatechin gallate (EGCG) disrupted existing G3BP1-cGAS complexes, blocking DNA-induced IFN production both in vivo and in vitro [92]. In a study examining IVD samples from IVDD patients, Tian et al. observed that mRNA levels in cGAS, STING, and NLRP3 were significantly elevated [43]. Follow-up experiments suggested that EGCG treatment could protect cell viability, apoptosis, cell cycle arrest, and inhibit pro-inflammatory factors release, presumably by attenuating the cGAS/STING/NLRP3 signaling pathway. These results suggest that EGCG may influence the cGAS-STING signaling pathway in IVDD. However, whether EGCG targets to prevent cGAS activation in IVDD still requires further investigation. In conclusion, small molecules and post-translational modifications aimed at cGAS carry substantial significance for modulating cGAS activity to better control over-activation of the STING signaling cascade. This offers exciting possibilities for their application in IVDD.

### Inhibitors of STING

STING is a primary signaling molecule in the DNA-sensing cGAS-STING signaling pathway, with its activation crucial in various signaling pathways. As such, efforts to develop STING inhibitors have been initiated, utilizing its crystal structure dynamics and regulatory factors. For instance, astin C, derived from Aster tataricus (a traditional Chinese medicinal plant), was identified by Li et al. as a potent bioactive compound. They confirmed that astin C could inhibit the cGAS-STING signaling pathway by specifically binding to the C-terminal activation pocket of STING [93]. Molecularly, STING palmitoylation promotes its clustering at the Golgi apparatus and subsequent recruitment of STING downstream signaling factors, making it a key factor enhancing STING activation [94]. Haag et al. demonstrated that nitrofuran derivatives (C-176, C-178, C-170, C-171, and H-151) could inhibit STING clustering induced by palmitoylation by covalently modifying the Cys91 residue of STING [95]. Tetrahydroisoquinoline acetic acid (compound 18) binds to STING by replacing cGAMP. After binding to Thr263 and Thr267 residues, compound 18 exhibited favorable slow dissociation kinetics and oral bioavailability, effectively inhibiting the cGAMP-dependent signaling pathway in an in vitro inhibitory manner [96]. On the other hand, butenolide heterodimer 13 can also inhibit the STING signaling pathway, although its specific target requires further verification and confirmation [97]. Concurrently, post-translational modifications of STING and downstream kinases have shown a similar inhibitory effect on STING activity. For example, amlexanox, an FDA-approved anti-inflammatory drug used to treat asthma and recurrent aphthous ulcers, has shown high affinity and specificity with TBK1. It can inhibit TBK1-induced phosphorylation of STING at Ser366, thus preventing STING activation [98, 99]. Notably, using amlexanox in sporadic AAD mice and human tissues has shown improvements in the structure of elastic fibers, preservation of the aortic structure, a reduction in the severity of rupture, slowdown of ECM degradation, and inhibition of STING and IRF3 phosphorylation. These findings suggest a potential preventive and delaying effect on aortic degeneration and the development of AAD [99, 100]. In addition, other TBK1 inhibitors, such as aminopyrimidines and GSK8612208, have been found to inhibit STING, offering potential benefits for STING-related diseases [101, 102]. Although these inhibitors have not been used in IVDD treatment, they could potentially alleviate the condition.

### Inhibitors upstream of cGAS-STING

Indeed, elevated levels of cytosolic DNA are associated with inflammation. Thus, eliminating cytosolic DNA might provide therapeutic benefits. As mentioned earlier, autophagy is an effective mechanism for removing damaged or cytoplasmic DNA. Therefore, issues such as autophagy defects can lead to an enhanced STING signaling pathway and exacerbate inflammatory phenotypes [103]. From this, it can be inferred that some level of autophagy activation can reduce the burden of clearing cytoplasmic DNA and have a therapeutic effect [104]. It has been found that the accumulation of DNA fragments in senescent cells of the activated cGAS-STING-NF-κB signaling pathway speeds up cellular aging. Simultaneously, the activation of autophagy, initiated by rapamycin, starts to play an active role, lowering DNA fragment levels and inhibiting the cGAS-STING-NF-κB signaling pathway [104]. Moreover, in the context of IVDD, Ren et al. found that Metformin treatment activated autophagy, which in turn degraded damaged DNA fragments, blocking the cGAS-STING signaling pathway and reducing the likelihood of downstream pro-inflammatory responses [85]. This offers a new avenue for regulating the upstream signaling of cGAS-STING in IVDD treatment.

### Inhibitors of downstream of cGAS–STING

Pathological conditions often result in increased levels of IFN-α and IFN-β [105–107]. This presents a compelling opportunity to target type-I IFN signaling in the treatment of infectious diseases, various cancers, and autoimmune diseases, including multiple sclerosis, SLE, and psoriasis [108, 109]. However, to our knowledge, no research has confirmed their roles in IVDD. In this scenario, regulating these factors may not alleviate the progression of IVDD. Nonetheless, current research in this area is insufficient, suggesting a direction for future studies.

## Conclusion and outlook

Following the onset of IVDD, NP cells situated at the center of the intervertebral disc undergo a phenotypic transition, resulting in the accumulation of DNA damage within the NP cells. Increasing evidence has shown that DNA damage triggers an inflammatory response in NP cells, which is closely linked to the degeneration of IVD [110, 111]. Recent studies focusing on the STING signaling pathway in IVDD have deepened our understanding of the role this pathway plays in IVDD. As outlined in this review, we have detailed the current knowledge of STING signaling and its protective effects on IVDD. Additionally, we summarized the latest advancements in our understanding of the regulatory mechanisms and signaling pathways of STING in apoptosis, autophagy, and pyroptosis. Finally, we have explored potential drugs that inhibit cGAS-STING signaling pathway activity, which may be beneficial for IVDD. Based on the current state of research, we propose the following suggestions for smoother development of follow-up studies: (1) The relationship of the cGAS-STING signaling pathway to various programmed cell death in IVDD should be extensively investigated. For instance, the connection between the cGAS-STING pathway and ferroptosis in IVDD should be one of the primary directions for future research. As a crucial constituent of the type I IFN response, the STING has been demonstrated to potentiate ferroptosis. Notably, research by Li and colleagues elucidates that erastin, a classic ferroptosis inducer, triggers STING accumulation in the mitochondria. This culminates in the production of reactive oxygen species, lipid peroxidation, and subsequent ferroptosis [112]. In the context of sepsis-induced cardiomyopathy, inhibiting Islet Cell Autoantigen 69 (ICA69), which consequently reduces STING expression, effectively curbs lipid peroxidation, ferroptosis, and the resultant inflammatory response [113]. Additionally, IRF3, a downstream effector of STING, has been implicated in lipid peroxidation and ferroptosis. This connection is notably observed in cardiac hypertrophy, where docosahexaenoic acid (DHA) counters microvascular endothelial cell dysfunction and cardiac impairment by obstructing ferroptosis [114]. During this mechanistic counteraction, DHA elevates IRF3 levels, thereby facilitating the transcription of SLC7A11 and concurrently reducing the production of arachidonate 12-lipoxygenase, resulting in ferroptosis inhibition [114]. In conclusion, these insights accentuate the role of the activated cGAS-STING signaling pathway in lipid peroxidation and ferroptosis, indicating potential therapeutic benefits in targeting this pathway to diminish the adverse effects mediated by ferroptosis. However, In IVDD, there is a scarcity of studies exploring the regulatory influence of cGAS-STING on ferroptosis. Therefore, this area demands comprehensive exploration in future research. (2) It is vital to elucidate the detailed mechanism of the cGAS-STING signaling pathway in IVDD. (3) The relationship between miRNAs and the STING signaling pathway warrants further exploration. For instance, it is interesting to consider whether miR-24–3p can reduce the inflammatory response in the hepatic I/R process by targeting the cGAS-STING signaling pathway, thereby mitigating apoptosis [115]. So far, there have been no reports on the regulatory effects of miRNAs on the cGAS-STING signaling pathway in IVDD, so further research in this area is necessary. These unresolved questions indicate that research into the cGAS-STING signaling pathway in IVDD can be deepened, offering a new direction for future research. In conclusion, this significance of review lies in providing a foundation for a deeper understanding of the role of the cGAS-STING signaling pathway in IVDD. It contributes to our comprehension of the unknown mechanisms of IVDD, and thus, is conducive to the early prevention and treatment of IVDD.

## Data Availability

This is a review and all data are included in the manuscript.
